# Ethnic disparities in pediatric appendicitis: the impact of hispanic ethnicity on presentation, complications, and postoperative outcomes

**DOI:** 10.1007/s00384-024-04598-8

**Published:** 2024-02-22

**Authors:** Charbel Chidiac, Olivia Liu, Rahul Gorijavolu, Daniel S. Rhee, Alejandro V. Garcia

**Affiliations:** 1 Department of Surgery, Johns Hopkins University School of Medicine, 1800 Orleans St Suite 7335, Baltimore, MD 21287 USA; 2 Johns Hopkins University School of Medicine, Baltimore, MD USA

**Keywords:** Appendectomy, Acute Appendicitis, Ethnic Disparities, Hispanic, Pediatric surgery

## Abstract

**Purpose:**

Our study investigates ethnic disparities in pediatric appendicitis, focusing on the impact of Hispanic ethnicity on presentation, complications, and postoperative outcomes.

**Methods:**

We conducted a retrospective analysis of pediatric patients undergoing appendectomy for acute appendicitis from 2015 to 2020 using the National Surgical Quality Improvement Program-Pediatric database. We compared 30-day postoperative complications, postoperative length of stay, and postoperative interventions between Hispanic and non-Hispanic White patients.

**Results:**

65,976 patients were included, of which 23,462 (35.56%) were Hispanic and 42,514 (64.44%) non-Hispanic White. Hispanic children were more likely to present to the hospital with complicated appendicitis (31.75% vs. 25.15%, *P* < 0.0001) and sepsis (25.22% vs. 19.02%, *P* < 0.0001) compared to non-Hispanic White. Hispanics had higher rates of serious complications (4.06% vs. 3.55%, *P* = 0.001) but not overall complications (5.37% vs. 5.09%, *P* = 0.12). However, after multivariate analysis, Hispanic ethnicity was not associated with an increased rate of serious postoperative complications (OR 0.93, CI 0.85–1.01, *P* = 0.088); it was associated with less overall complications (OR 0.88, CI 0.81–0.96, *P* = 0.003) but a longer postoperative length of stay (OR 1.09, CI 1.04–1.14, *P* < 0.0001).

**Conclusion:**

Hispanic children are more likely to present with complicated appendicitis, contributing to increased postoperative complications. Notably, upon adjustment for the impact of complicated appendicitis, our findings suggest potentially favorable outcomes for Hispanic ethnicity. This emphasizes the need to understand delays in presentation to improve outcomes in the Hispanic population.

## Introduction

Appendicitis is the most common pediatric surgical emergency, accounting for nearly 30% of all emergency room visits for abdominal pain [1, 2]. Timely diagnosis and treatment are imperative in mitigating the risk of perforation and its associated complications. Delayed diagnosis is associated with an increased hospital length of stay, perforation, and risk of multiple operative interventions .

Racial and ethnic disparities have been well described in the field of surgery, whether in abdominal, cardiac, oncologic, or trauma, with clear associations between minority status and poor surgical outcomes, despite advancements in perioperative care [3–7]. In children and in adults undergoing appendectomy, Black patients experience a higher rate of postoperative complications and longer length of stay than White patients [8–11]. However, differences in surgical outcomes between Hispanic and non-Hispanic patients are not as widely studied but are known to exist: *Eguia et al.* showed that Hispanics undergoing low- to high-risk surgery have worse outcomes compared to non-Hispanic Whites [12].

Our study aims to compare the initial presentation and postoperative outcomes of Hispanic and non-Hispanic children surgically treated for acute appendicitis in order to understand where interventions should be focused to mitigate these disparities.

## Methods

### Data source

Data were extracted from the American College of Surgeons (ACS) National Surgical Quality Improvement Program- Pediatric (NSQIP-P) from 2015 to 2020. The NSQIP-P is a multi-institutional program that contains risk-adjusted safety outcomes data for surgical patients between 0 and 18 years of age. Reported data included demographics, comorbidities, laboratory values, and outcomes within 30 days after surgery. NSQIP-P data are prospectively collected by trained surgical reviewers at different hospitals, and several methods are in place to ensure high data quality including standardized data collection, data validation and verification, site visits and audits, risk adjustment, and continuous improvement efforts. Our study adheres to the Strengthening the Reporting of Observational studies in Epidemiology (STROBE) checklist for cohort studies.

### Study cohort

Children aged 18 years or less were included in the analysis if they underwent either open or laparoscopic appendectomy (primary Current Procedural Terminology [CPT] 44,950, 44,960, 44,970) and had a postoperative diagnosis confirming acute appendicitis (International Classification of Diseases [ICD], 9th edition codes 540, 540.0, 540.1, 540.9/ ICD-10 code K35 and its sub-classifications e.g., K35.32, K35. 89). Patients were classified as Hispanic of any race or non-Hispanic White. Patients were excluded if they had an appendectomy performed outside their initial hospital presentation. Non-Hispanic Black, Asian, other races and unknown races of children were excluded from the study.

### Predictor variables

The primary variable of this study was patient ethnicity (Hispanic vs. non-Hispanic White). Demographic and patient factors included age, gender, weight, American Society of Anesthesiologists (ASA) physical status classification (ASA 1: Normal healthy patient, ASA 2: Patient with mild systemic disease, ASA 3: Patient with severe systemic disease, ASA 4: Patient with severe systemic disease that is a threat to life), neurologic impairment (including impaired cognitive status, cerebral palsy, seizure disorder, structural central nervous system defect, and neuromuscular disorder), asthma, bleeding disorder, cardiac risk factors as defined by the NSQIP (minor, major and severe), wound classification (classified as clean, clean/contaminated, contaminated, dirty/infected), preoperative sepsis (none, systemic inflammatory response syndrome [SIRS], sepsis and septic shock), classification of operation posting (elective vs. urgent vs. emergent), surgical approach (open vs. laparoscopic), and severity of appendicitis (complicated vs. uncomplicated). Appendicitis was defined as complicated if one of the following criteria was met: the presence of a visible hole in the appendix, the existence of a fecalith in the peritoneal cavity outside of the appendix, the identification of an abscess, or the observation of diffuse fibrinopurulent exudate in the peritoneal cavity.

### Outcome variables

The primary outcomes were the development of one or more serious postoperative complications, any postoperative complications, and the postoperative length of stay. A serious complication was defined as any of the following: organ or space surgical site infection, wound disruption or dehiscence, bleeding that required transfusion, postoperative sepsis or septic shock and reoperation [13–15]. Any postoperative complication was defined as ≥ 1 of following: serious complication, superficial surgical site infection, deep incisional surgical site infection, pneumonia, urinary tract infection, unplanned intubation, and *Clostridioides difficile* (C. diff) infection.

Secondary outcomes included placement of percutaneous drainage, peripherally inserted central catheter (PICC) placement, parenteral nutrition, post-op fever, and discharge on oral or parenteral antibiotics. These outcomes were chosen as they relate to the severity of the appendicitis.

### Missing data analysis

We excluded patients with missing data on race and ethnicity. For the remaining variables, missing data were determined to be missing completely at random using Little’s missing completely at random test. We performed multiple imputations using the Markov Chain Monte Carlo method to reduce bias and preserve sample size. The postoperative complication variables were complete with no missing values.

### Statistical analysis

Continuous variables are reported as median with interquartile range (IQR), whereas categorical variables are reported as frequencies and percentages. Two-sided t-tests or Wilcoxon’s rank-sum tests were used for continuous variables while Chi-squared or Fisher exact tests were used for categorical ones.

Postoperative serious and overall complications were compared between Hispanics and non-Hispanic Whites using multivariate logistic models. Postoperative length of stay (LOS) was compared using a multivariate generalized linear model. A variety of potential variables that could be confounders (age at operation, sex, ethnicity, weight, preoperative sepsis, presentation with complicated appendicitis, and surgical approach) were selected. Forward stepwise regression to reduce AIC (Akaike Information Criterion) was used to identify the confounder to be included in the final model. The primary predictor variable (ethnicity) was not forced into the model.

We further conducted a sub analysis by categorizing patients into groups based on the presence of complicated or uncomplicated appendicitis. We then compared the 30-day postoperative complication rates as well as postoperative intervention between Hispanic and non-Hispanic White children in each group separately. We chose this segregation because patients with complicated appendicitis often follow a distinct clinical course compared to those without complications.

A *p*-value of less than 0.05 was considered statistically significant. All statistical analysis was performed using R Statistical software (version RStudio 2022.07.2 + 576 “Spotted Wakerobin”).

## Results

**Fig. 1:**
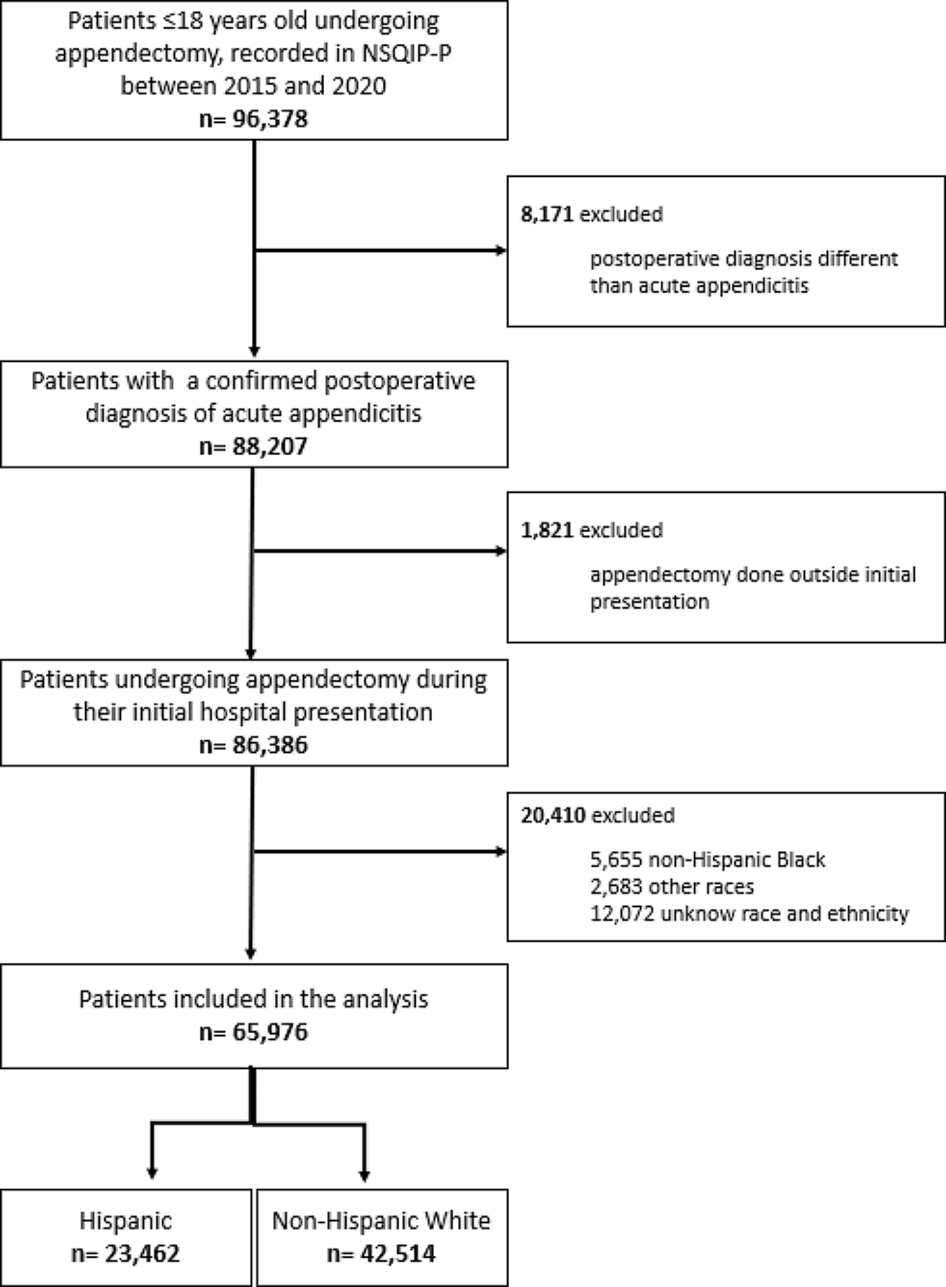
Study inclusion/exclusion criteria and sample sizes

A total of 96,378 children, aged 18 years and younger, who underwent appendectomy were identified in the NSQIP-P database from 2015 to 2020. Patients with a postoperative diagnosis differing from acute appendicitis (*n* = 8,171) and those undergoing appendectomy outside their initial presentation (*n* = 1,821) were excluded. Additionally, 5,655 non-Hispanic Black children, 2,683 of other races, and 12,072 with unknown race and ethnicity were excluded. The final analysis included 65,976 children, among whom 23,462 (35.56%) identified as Hispanic and 42,514 (64.44%) as non-Hispanics White (Fig. 1). Demographic and preoperative characteristics of patients by ethnicity are presented in Table 1. The rate of complicated appendicitis was higher in Hispanic patients compared to non-Hispanic White patients (31.75% vs. 25.15%, *P* < 0.0001). Hispanics were less likely to undergo laparoscopic procedures than non-Hispanic White ones (94.07% Hispanic vs. 95.13% non-Hispanic White, *P* < 0.0001).

**Table 1:** Demographic and preoperative characteristics of children undergoing appendectomy for acute appendicitis by ethnicity

	Total, *n* = 65,976 (%)	Hispanic, *n* = 23,462 (%)	Non-Hispanic white, *n* = 42,514 (%)	*P*-value
Age (Years) (mean +/-SD)	11.3 +/ 3.7	10.7 +/- 3.8	11.5 +/- 3.7	**< 0.0001**
Female	26,045 (39.48)	9,067 (38.65)	16,978 (39.94)	**0.002**
Weight (Pounds) (mean +/-SD)	98.6 +/- 42.8	97.0 +/- 43.3	99.4 +/- 42.2	**< 0.0001**
Neurologic impairment	1,779 (2.70)	635 (2.71)	1,144 (2.69)	0.93
Asthma	2,693 (4.08)	940 (4.01)	1,753 (4.12)	0.48
Bleeding disorder	495 (0.75)	163 (0.69)	332 (0.78)	0.24
ASA Class				0.48
1	32,138 (48.71)	11,383 (48.52)	20,755 (48.82)	
2	30,902 (46.84)	11,005 (46.91)	19,897 (46.80)	
3	2,846 (4.31)	1,037 (4.42)	1,809 (4.26)	
4	90 (0.14)	37 (0.16)	53 (0.12)	
Cardiac Risk factors				0.07
Minor	526 (0.80)	172 (0.73)	354 (0.83)	
Major	328 (0.50)	99 (0.42)	229 (0.54)	
Severe	45 (0.07)	13 (0.06)	32 (0.08)	
Wound classification				**< 0.0001**
Clean	1,377 (2.09)	428 (1.82)	949 (2.23)	
Clean/contaminated	10,144 (15.38)	3,287 (14.01)	6,857 (16.13)	
Contaminated	37,042 (56.14)	12,868 (54.85)	24,174 (56.86)	
Dirty/infected	17,413 (26.39)	6,879 (29.32)	10,534 (24.78)	
Preoperative sepsis				**< 0.0001**
None	30,664 (46.48)	9,482 (40.41)	21,182 (49.83)	
SIRS	21,257 (32.22)	8,039 (34.26)	13,218 (31.09)	
Sepsis	14,002 (21.22)	5,916 (25.22)	8,086 (19.02)	
Septic shock	52 (0.08)	25 (0.11)	27 (0.06)	
Case type				**< 0.0001**
Elective	6,750 (10.23)	2,168 (9.24)	4,582 (10.78)	
Urgent	24,878 (37.71)	8,345 (35.57)	16,533 (38.89)	
Emergent	34,348 (52.06)	12,949 (55.19)	21,399 (50.33)	
Complicated Appendicitis	18,144 (27.50)	7,450 (31.75)	10,694 (25.15)	**< 0.0001**
Laparoscopic approach	62,512 (94.75)	22,070 (94.07)	40,442 (95.13)	**< 0.0001**

ASA American Society of Anesthesiologists; SD Standard deviation; SIRS Systemic inflammatory response syndrome

*P*-values in bold indicate statistical significance

Postoperative complications in Hispanic and non-Hispanic White patients are demonstrated in Table 2. Hispanics were more likely to experience serious complications compared to Whites (4.06% vs. 3.55%, *P* = 0.001), however, there was no difference in rates of overall complications between them (5.37% for Hispanics vs. 5.09% for Whites, *P* = 0.12). Of the 14 postoperative complications included in our analysis, Hispanics had a significantly higher incidence for 3 (organ/space surgical site infection, Clostridium difficile infection and rate of reoperation) while Whites only had a higher incidence of pneumonia. In addition, the rate of postoperative emergency department (ED) visits related to the appendectomy was higher in Hispanics compared to Whites (8.78% vs. 6.82%, *P* < 0.0001).

**Table 2:** 30-day postoperative outcomes after appendectomy by patient ethnicity

	Total, *n* = 65,976 (%)	Hispanic, *n* = 23,462 (%)	Non-Hispanic white, *n* = 42,514 (%)	*P*-value
**Overall complications**	3,422 (5.19)	1,260 (5.37)	2,162 (5.09)	0.12
**Serious complications**	2,463 (3.73)	952 (4.06)	1,511 (3.55)	**0.001**
**Superficial SSI**	683 (1.04)	232 (0.99)	451 (1.06)	0.40
**Deep incisional SSI**	90 (0.14)	36 (0.15)	57 (0.13)	0.60
Organ/space SSI*	1,932 (2.92)	744 (3.17)	1,188 (2.79)	**0.006**
Wound disruption*	27 (0.04)	9 (0.04)	18 (0.04)	0.97
**Pneumonia**	63 (0.10)	13 (0.06)	50 (0.12)	**0.02**
**Urinary tract infection**	62 (0.09)	28 (0.12)	34 (0.08)	0.15
**Unplanned intubation**	8 (0.01)	3 (0.01)	5 (0.01)	1
**Seizure**	10 (0.02)	2 (0.01)	8 (0.02)	0.51
Bleeding*	65 (0.10)	30 (0.13)	35 (0.08)	0.09
Sepsis*	196 (0.30)	79 (0.34)	117 (0.28)	0.19
Septic shock*	23 (0.03)	11 (0.05)	12 (0.03)	0.31
***Clostridioides difficile*** **infection**	216 (0.32)	56 (0.25)	160 (0.4)	**0.003**
Rate of reoperation*	621 (0.94)	245 (1.05)	376 (0.88)	**0.0009**

**SSI** surgical site infection

* These complications were considered serious complications

*P*-values in bold indicate statistical significance

In addition, Hispanic patients experienced longer postoperative length of stay (for Hispanics vs. Whites: mean 1.56 vs. 1.02 days; median 1 vs. 1 day; interquartile range [IQR] 0–2 vs. 0–1; *P* < 0.0001) and underwent more postoperative interventions including percutaneous drainage placement (3.65% vs. 3.12%, *P* = 0.0002), PICC placement (2.57% vs. 2.28%, *P* = 0.02), postoperative imaging with computed tomography (CT) (5.32% vs. 4.67%, *P* = 0.0002) or ultrasound (4.84% vs. 4.25%, *P* = 0.0005). Hispanic children were also more likely to develop postoperative fever (2.65% vs. 1.81%, *P* < 0.0001) and to be discharged on oral antibiotics (25.60% vs. 20.29%, *P* < 0.0001) (Table 3).

**Table 3:** Postoperative length of stay and interventions after pediatric appendectomy by patient ethnicity

	Total, *n* = 65,976 (%)	Hispanic, *n* = 23,462 (%)	Non-Hispanic white, *n* = 42,514 (%)	*P*-value
**Post-op length of stay**	1 [0–2]	1 [0–2]	1 [0–1]	**< 0.0001**
Complicated appendicitis	3 [2–4]	3 [2–5]	3 [2–4]	**< 0.0001**
Uncomplicated appendicitis	1 [0–1]	1 [0–1]	1 [0–1]	0.67
**Post-operative pathologic finding**				**< 0.0001**
Normal appendix	980 (1.49)	267 (1.14)	713 (1.68)	
Appendicitis	63,126 (95.68)	22,609 (96.36)	40,517 (95.30)	
Other appendiceal disease	1799 (2.73)	562 (2.40)	1237 (2.91)	
**Post-operative imaging**				
US	2,943 (4.46)	1,135 (4.84)	1,808 (4.25)	**0.0005**
CT	3,232 (4.90)	1,248 (5.32)	1,984 (4.67)	**0.0002**
MRI	199 (0.30)	68 (0.29)	131 (0.31)	0.74
**Fever**	1,390 (2.11)	621 (2.65)	769 (1.81)	**< 0.0001**
**Percutaneous drainage**	2183 (3.31)	857 (3.65)	1,326 (3.12)	**0.0002**
**PICC placement**	1,574 (2.39)	604 (2.57)	970 (2.28)	**0.02**
**Parenteral nutrition**	1,136 (1.72)	427 (1.82)	709 (1.67)	0.16
**Oral antibiotics at discharge**	14,631 (22.18)	6,006 (25.60)	8,625 (20.29)	**< 0.0001**
**Parenteral antibiotics at discharge**	707 (1.07)	157 (0.67)	550 (1.29)	**< 0.0001**
**Rate of readmission**	1,840 (2.79)	667 (2.84)	1,173 (2.76)	0.55
**Rate of ED visits**	4,959 (7.51)	2,059 (8.78)	2,900 (6.82)	**< 0.0001**

**CT** computerized tomography; **ED** Emergency department; **MRI** Magnetic Resonance Imaging; **PICC** Peripherally inserted central catheter; **US** Ultrasound

*P*-values in bold indicate statistical significance

After accounting for potential confounding factors on multivariate analysis (Table 4) including age, preoperative sepsis, presentation with a complicated appendicitis, and surgical approach, the association of Hispanic ethnicity with increased incidence of serious complications lost its significance (adjusted odds ratio [OR] 0.93, 95% confidence interval [CI] 0.85–1.01, *P* = 0.088) while its association with increased postoperative LOS maintained it (OR 1.09; IQR 1.04–1.14; *P* < 0.0001). In addition, Hispanic ethnicity was associated with a decreased incidence of overall complications (OR 0.88; 95% CI 0.81–0.96; *P* = 0.004) when accounting for how patients present. Among the various factors studied, complicated appendicitis emerged as the primary cause of postoperative serious complications (OR 6.54; 95% CI 5.77–7.41; *P* < 0.0001), overall complications (OR 5.55; 95% CI 4.95–6.23; *P* < 0.0001), and an extended length of stay (OR 10.74, 95% CI 10.08–11.43; *P* < 0.0001).

**Table 4:** Multivariate analysis of predictors of serious and overall complications and post-operative length of stay in children undergoing appendectomy for acute appendicitis

	Serious complications	*P*-value		Overall complications	*P*-value		Post-operative length of stay	*P*-value
**Hispanic ethnicity**	0.93 [0.85–1.01]	0.088		0.88 [0.81–0.96]	**0.004**		1.09 [1.04–1.14]	**< 0.0001**
**Age (in years)**	1 [0.99–1.01]	0.99		1 [0.99–1.01]	0.81		0.97 [0.97–0.98]	**< 0.0001**
**SIRS**	0.94 [0.81–1.1]	0.45		0.95 [0.83–1.08]	0.42		0.97 [0.93–1.02]	0.26
**Sepsis**	2.43 [2.17–2.73]	**< 0.0001**		2.35 [2.11–2.62]	**< 0.0001**		3.24 [3.03–3.48]	**< 0.0001**
**Complicated appendicitis**	6.54 [5.77–7.41]	**< 0.0001**		5.55 [4.95–6.23]	**< 0.0001**		10.74 [10.08–11.43]	**< 0.0001**
**Laparoscopic approach**	0.65 [0.56–0.76]	**< 0.0001**		0.68 [0.59–0.79]	**< 0.0001**		0.67 [0.61–0.74]	**< 0.0001**

**SIRS** Systemic inflammatory response syndrome

Values are presented as odds ratio [95% confidence intervals]

*P*-values in bold indicate statistical significance

**Fig. 2:**
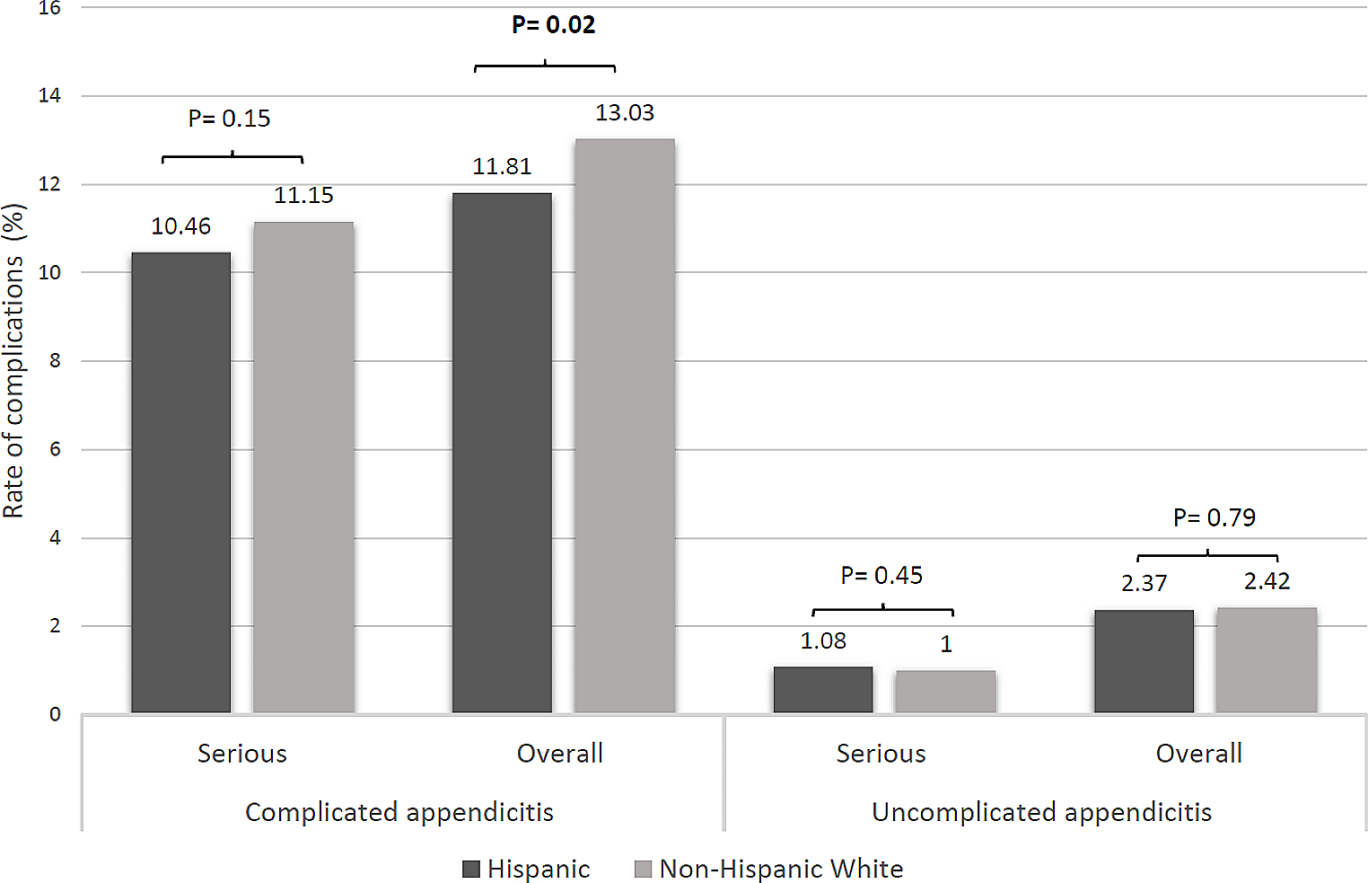
Rate of serious and overall complications by ethnicity in complicated and uncomplicated appendicitis separately. Chi-Square tests were used were used to compare the rate of complications between Hispanic children and non-Hispanic White children. *P*-values in bold indicate statistical significance

After segregating patients by complicated and uncomplicated appendicitis, in the uncomplicated group, no difference in overall or serious complications was seen between Hispanic and non-Hispanic White children (overall: 2.37% vs. 2.42% respectively, *P* = 0.79; serious: 1.08% vs.1.00%,*P* = 0.45) (Table 5; Fig. 2). However, Hispanic patients had a higher rate of ED visits within 30-days compared non-Hispanic White ones (7.16% vs. 5.24%, *P* < 0.0001). On the other hand, in the complicated group, Hispanic children experienced fewer overall complications than non-Hispanic White children (11.81% vs. 13.03% respectively, *P* = 0.02) but similar serious complications (10.46% vs. 11.15%, *P* = 0.15) (Table 5). Additionally, they experience more postoperative fever (7.25% vs. 5.83%, *P* = 0.0004) and are discharged more on oral antibiotics (62.69% vs. 60.77%, *P* = 0.009) (Table 6).

**Table 5:** 30-day postoperative outcomes after appendectomy by patient ethnicity segregated by uncomplicated and complicated appendicitis

	Uncomplicated				Complicated		
	Hispanic *n* = 16,012 (%)	Non-Hispanic White *n* = 31,820 (%)	*P*-value		Hispanic *n* = 7,450 (%)	Non-Hispanic White *n* = 10,694 (%)	*P*-value
**Overall complications**	380 (2.37)	769 (2.42)	0.79		880 (11.81)	1,393 (13.03)	**0.02**
**Serious complications**	173 (1.08)	319 (1.00)	0.45		779 (10.46)	1,192 (11.15)	0.15
**Superficial SSI**	504 (1.05)	166 (1.04)	0.83		66 (0.89)	113 (1.06)	0.29
**Deep incisional SSI**	14 (0.09)	25 (0.08)	0.88		22 (0.30)	32 (0.30)	1
Organ/space SSI*	96 (0.60)	180 (0.57)	0.69		648 (8.70)	1,008 (9.43)	0.10
Wound disruption*	3 (0.02)	7 (0.02)	1		6 (0.08)	11 (0.1)	0.81
**Pneumonia**	6 (0.04)	23 (0.07)	0.21		7 (0.09)	27 (0.25)	**0.02**
**Urinary tract infection**	17 (0.11)	30 (0.09)	0.81		11 (0.15)	4 (0.04)	**0.02**
**Unplanned intubation**	0 (0)	2 (0.01)	0.80		3 (0.04)	3 (0.03)	0.98
**Seizure**	1 (0.01)	3 (0.01)	1		1 (0.01)	5 (0.05)	0.42
Bleeding*	15 (0.09)	15 (0.05)	0.08		15 (0.20)	20 (0.19)	0.96
Sepsis*	36 (0.22)	41 (0.13)	**0.02**		43 (0.58)	76 (0.71)	0.32
Septic shock*	3 (0.02)	2 (0.01)	0.43		8 (0.11)	10 (0.09)	0.96
***Clostridioides difficile*** infection*	25 (0.16)	76 (0.25)	0.07		31 (0.44)	84 (0.83)	**0.002**
Rate of reoperation*	70 (1.17)	139 (1.04)	0.43		175 (5.83)	237 (5.12)	0.20

**SSI** surgical site infection

* These complications were considered serious complications

*P*-values in bold indicate statistical significance

**Table 6:** Postoperative length of stay and interventions after pediatric appendectomy by patient ethnicity segregated by uncomplicated and complicated appendicitis

	Uncomplicated				Complicated		
	Hispanic *n* = 16,012 (%)	Non-Hispanic White *n* = 31,820 (%)	*P*-value		Hispanic *n* = 7,450 (%)	Non-Hispanic White *n* = 10,694 (%)	*P*-value
**Post-op length of stay**	1 [0–1]	1 [0–1]	0.67		3 [2-5]	3[2-4]	**< 0.0001**
**Post-operative pathologic finding**			**< 0.0001**				0.90
Normal appendix	240 (1.50)	676 (2.12)			28 (0.38)	37 (0.35)	
Appendicitis	15,277 (95.4)	29,997 (94.28)			7,334 (98.43)	10,518 (98.35)	
Other appendiceal disease	479 (2.99)	1,106 (3.48)			83 (1.11)	131 (1.22)	
**Post-operative imaging**							
US	9,812 (61.27)	18,359 (57.7)	**< 0.0001**		4,285 (57.51)	5,595 (52.32)	**< 0.0001**
CT	2,610 (16.3)	4,440 (13.95)	**< 0.0001**		1,720 (23.08)	2,110 (19.73)	**< 0.0001**
MRI	427 (2.67)	770 (2.42)	0.11		206 (2.76)	241 (2.25)	**0.03**
**Fever**	81 (0.51)	146 (0.46)	0.69		540 (7.25)	623 (5.83)	**0.0004**
**Percutaneous drainage**	95 (0.59)	216 (0.68)	0.30		762 (10.23)	1,110 (10.38)	0.76
**PICC placement**	41 (0.26)	89 (0.28)	0.71		563 (7.56)	881 (8.24)	0.10
**Parenteral nutrition**	34 (0.21)	64 (0.2)	0.88		393 (5.27)	645 (6.03)	**0.03**
**Oral antibiotics at discharge**	1,336 (8.34)	2,126 (6.68)	**< 0.0001**		4,671 (62.69)	6,499 (60.77)	**0.009**
**Parenteral antibiotics at discharge**	19 (0.12)	310 (0.97)	**< 0.0001**		138 (1.85)	240 (2.24)	0.08
**Rate of readmission**	284 (1.77)	591 (1.86)	0.54		486 (6.52)	794 (7.42)	**0.02**
**Rate of ED visits**	1,147 (7.16)	1,666 (5.24)	**< 0.0001**		912 (12.24)	1,234 (11.54)	0.16

**CT** computerized tomography; **ED** Emergency department; **MRI** Magnetic Resonance Imaging; **PICC** Peripherally inserted central catheter; **US** Ultrasound

*P*-values in bold indicate statistical significance

## Discussion

Our study aims to better understand ethnic disparities in perioperative outcomes of children undergoing appendectomy. We found that ethnic disparities exist in the context of acute appendicitis in children, especially in preoperative presentation; Hispanic children exhibited a higher likelihood of presenting with complicated appendicitis compared to their non-Hispanic White counterparts. Consequently, this contributed to an elevated overall complication rate for all acute appendicitis cases within the Hispanic population. However, upon careful adjustment for multiple factors, including the presence of complicated appendicitis, our analysis indicates that there is no increased risk of complications in the Hispanic population. Instead, the observed disparities appear to be predominantly driven by delayed presentation and the subsequent development of complicated appendicitis.

In contrast to non-Hispanic White children, Hispanic children exhibited a higher frequency of surgical drains, open surgeries, postoperative fevers, postoperative imaging (including CT scans and ultrasounds), prolonged length of stay, and an increased rate of postoperative emergency department visits. These findings are consistent with the higher incidence of complicated appendicitis and appendiceal perforation previously documented in people of Hispanic ethnicity [16–18]. Studies suggest that Hispanic patients present with a more severe form of appendicitis, which is then associated with worse outcomes and the further perpetuation of disparity [19, 20]. This difference could be reflective of the social determinants of health, which include factors such as economic stability, neighborhood and physical environment, education, community, and social context, as well as the health care system. Barriers to care in Hispanic patients such as limited English language proficiency, lower health insurance rates and lack of transportation in conjunction with lower health literacy are factors that may contribute to the delay of care and more complex presentation of appendicitis [20–23]. Studies have also shown that rates of pediatric appendiceal perforation are higher in Medicaid and uninsured patients, as parents of these children are likely delaying care due to financial factors and failure to recognize symptoms [16].

Parental health literacy is a strong predictor of childhood health, and thus, it is critical to deliver quality health education to parents and caregivers to aid in early intervention and improve postoperative outcomes [24]. Parental knowledge of the symptoms can influence the timing of care that children receive and adherence to discharge instructions [25]. Providing parents with comprehensive education on monitoring for negative symptoms and the urgency of seeking medical care will likely improve outcomes. Additionally, Spanish-speaking patients face language barriers [23, 26]. Limitations in English proficiency have been found to be associated with longer postoperative length of stay, decreased patient satisfaction, and higher rates of adverse events [27–29]. Language barriers are also associated with decreased discharge instruction comprehension and increased preventable emergency department visits [30, 31]. Interventions to reduce the language barrier and improve caregiver-physician communication are imperative in improving pediatric appendicitis outcomes.

Our study is not without limitations. First, it is a retrospective analysis. Second, social determinants of health variables, including socioeconomic status, insurance status, distance to care, and language preference as well as hospital-level factors such as location, academic status of the hospital were not recorded in the database. Notably, some outcomes such as PICC placement, starting TPN and discharge on antibiotics might depend on institutional practice and surgeon preference. Nevertheless, our large sample size provides a clear picture of macroscopic issues that can then be further investigated through more focused research. Finally, it is important to consider the clinical relevance of the observed statistical differences. In our study, several observed differences reached statistical significance due to our large sample size, such as a half-day postoperative length of stay, PICC placement, and percutaneous drainage among others. Nevertheless, the generalizability of our study results is strengthened by the utilization of a national database and the inclusion of a substantial sample size.

In conclusion, Hispanic children were more likely to experience postoperative complications and an increased length of stay compared to non-Hispanic White children. This is to be perceived as being due to their higher rate of complicated appendicitis at presentation and not to any imbalance in care management. Therefore, the main issue lies prior to hospital presentations, and efforts should be made to understand what factors, including social determinants of health, access to care, language barrier and hospital level factors, aggravate this situation. This insight will inform targeted interventions and policies aimed at mitigating disparities and enhancing equitable access to healthcare across diverse populations.

### Disclosure

The American College of Surgeons National Surgical Quality Improvement Program and the hospitals participating in the ACS NSQIP Pediatric are the sources of the data used herein; they have not verified and are not responsible for the statistical validity of the data analysis or the conclusions derived by the authors.

## Data Availability

The American College of Surgeons National Surgical Quality Improvement Program- Pediatrics (NSQIP-P) database can be accessed by requesting the database from the American College of Surgeons. The link to the participant use request form is the following: https://www.facs.org/quality-programs/data-and-registries/acs-nsqip/participant-use-data-file/participant-use-request-form/.

## Abbreviations

ACS: American College of Surgeons
AIC: Akaike Information Criterion
ASA: American Society of Anesthesiologists
C. diff: *Clostridioides difficile*
CPT: Current Procedural Terminology
CT: Computed tomography
ED: Emergency department
ICD: International Classification of Diseases
IQR: Interquartile range
LOS: Length of stay
NSQIP-P: National Surgical Quality Improvement Program- Pediatric
PICC: Peripherally inserted central catheter
SIRS: Systemic inflammatory response syndrome
STROBE: Strengthening the Reporting of Observational studies in Epidemiology
